# A quantitative metric for organic radical stability and persistence using thermodynamic and kinetic features†

**DOI:** 10.1039/d1sc02770k

**Published:** 2021-09-06

**Authors:** Shree Sowndarya S. V., Peter C. St. John, Robert S. Paton

**Affiliations:** Department of Chemistry, Colorado State University Fort Collins CO 80523 USA robert.paton@colostate.edu; Biosciences Center, National Renewable Energy Laboratory Golden CO 80401 USA peter.stjohn@nrel.gov

## Abstract

Long-lived organic radicals are promising candidates for the development of high-performance energy solutions such as organic redox batteries, transistors, and light-emitting diodes. However, “stable” organic radicals that remain unreactive for an extended time and that can be stored and handled under ambient conditions are rare. A necessary but not sufficient condition for organic radical stability is the presence of thermodynamic stabilization, such as conjugation with an adjacent π-bond or lone-pair, or hyperconjugation with a σ-bond. However, thermodynamic factors alone do not result in radicals with extended lifetimes: many resonance-stabilized radicals are transient species that exist for less than a millisecond. Kinetic stabilization is also necessary for persistence, such as steric effects that inhibit radical dimerization or reaction with solvent molecules. We describe a quantitative approach to map organic radical stability, using molecular descriptors intended to capture thermodynamic and kinetic considerations. The comparison of an extensive dataset of quantum chemical calculations of organic radicals with experimentally-known stable radical species reveals a region of this feature space where long-lived radicals are located. These descriptors, based upon maximum spin density and buried volume, are combined into a single metric, the radical stability score, that outperforms thermodynamic scales based on bond dissociation enthalpies in identifying remarkably long-lived radicals. This provides an objective and accessible metric for use in future molecular design and optimization campaigns. We demonstrate this approach in identifying Pareto-optimal candidates for stable organic radicals.

## Abstract 2

Molecular descriptors encoding kinetic and thermodynamic stabilization capture the difference between transient and persistent organic radicals.

## Graphical abstract

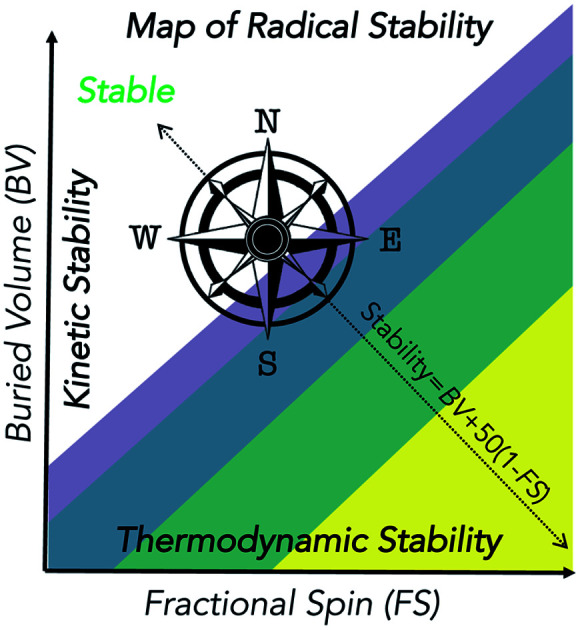

## Introduction

From the initial discovery of free radicals to their becoming textbook chemistry, it has been emphasized that a molecule containing an unpaired electron (*i.e.*, a free radical) is likely very reactive. Over the years, however, organic chemists have addressed the importance of studying organic radicals' stability.^1^ Since Gomberg's discovery that the triphenylmethyl radical does not dimerize to form hexaphenylethylene, the search for stable radicals has intensified.^2^ In this field, the term “stable radical” is proposed to refer to a compound that is unreactive enough so that “the pure radical can be handled and stored in the lab with no more precautions than would be used for the majority of commercially available organic chemicals”.^3^ Ingold also classified radicals according to their lifetime; transient radicals are those with a half-life less than a millisecond, while persistent radicals are those with a longer half-life.^3^ In this context, a stable radical able to be stored in air can be seen as an example of extreme persistence.

**Fig. 1:**
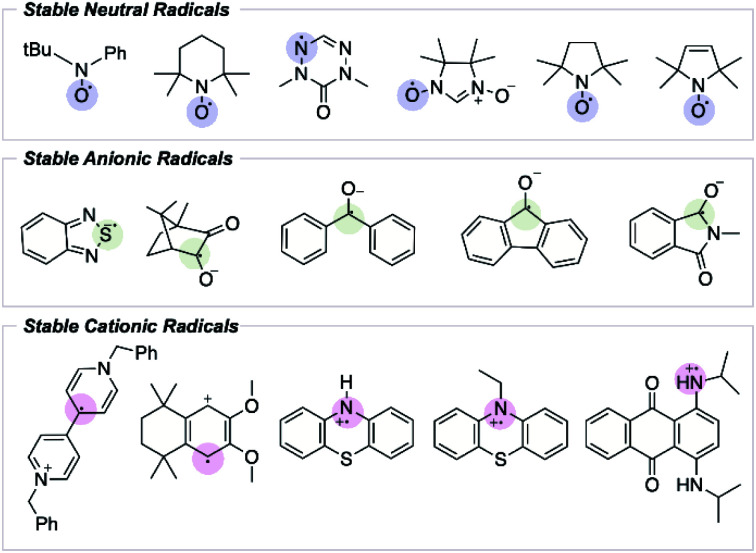
Experimentally confirmed stable organic radicals.

Most radicals are highly reactive and transient. In contrast, stable radical species possess unique electronic and reduction–oxidation (redox) properties that have spurred interest as potential materials in energy storage and energy conversion devices.^4–7^ The electronic and steric features influencing radical stability have been well studied; however, predicting a radical's lifetime remains challenging, and only a handful of experimentally stable organic radical species have been reported (Fig. 1).^8,9^ While it may be possible to extend the lifetime of an already persistent radical through structural fine-tuning, the discovery of new stable radical functionalities has been restricted by the absence of a quantitative description of radical stability that extends beyond simple thermodynamic considerations, and that can be used predictively.

Comparative studies of radical stability have focused mainly on aspects of thermodynamic stabilization. For example, these effects in organic radicals have been quantified by defining a radical stabilization energy (RSE) scale.^10,11^ The RSE for a carbon-centered radical R˙ is defined by the difference between C–H bond dissociation energies (BDEs) of methane and R–H (an isodesmic H-atom transfer reaction).^12^ Weaker R–H bonds give rise to increasingly positive RSE values that reflect increasing thermodynamic stabilization of the R˙ radical. Alternative RSE schemes have been proposed to minimize the differential contributions of C–H bond polarity in these comparisons, such as by comparing the C–C BDEs of CH_3_–CH_3_*vs.* R–R.^13,14^ Additionally, for radicals centered on oxygen, nitrogen, or sulfur atoms, molecules such as H_2_O, NH_3_, and H_2_S define separate RSE scales.^15–17^

RSE schemes have been instrumental in enabling quantitative comparisons of radical stabilization that can be performed computationally, using composite *ab initio* methods or lower-cost density functional theory (DFT) calculations. However, several fundamental issues limit the usefulness of RSE values for discovering new stable radicals. Firstly, persistence is a kinetic phenomenon relating to rates of reactivity and influenced by steric stabilization, while RSEs are thermodynamic in origin. For example, although electron delocalization provides thermodynamic stabilization and an RSE value of 14.6 kcal mol^−1^, the benzyl radical is a transient species with a lifetime of less than a millisecond.^18^ Secondly, the referencing of RSE values with a specific bond-type (*e.g.*, C–H) does not allow for a universal comparison of different radical types, such as carbon-centered *vs.* oxygen-centered radicals.^19^ At the same time, quantitative metrics to describe kinetic persistence are still in their infancy, and no general method is yet available. To address these limitations, we propose a new metric for quantifying radical persistence, able to identify and predict stable organic radical structures. Its development incorporates kinetic and thermodynamic considerations, and the metric is generally applicable to carbon- or heteroatom (N, O, S) centered radicals. We assembled a sizeable computational database of organic radicals and have identified two DFT-derived features derived from the quantum mechanical spin density distribution and the molecular geometry that can be used to chart thermodynamic and kinetic variability. With this approach, we can cluster radicals into distinct regions and identify the region of this feature space where experimentally validated stable radicals reside.^20^ The resulting quantitative metric for stable radicals provides a route to high-throughput searches and generative design efforts for stable organic radicals precisely tuned for emerging energy material applications.

## Results and discussion

**Fig. 2:**
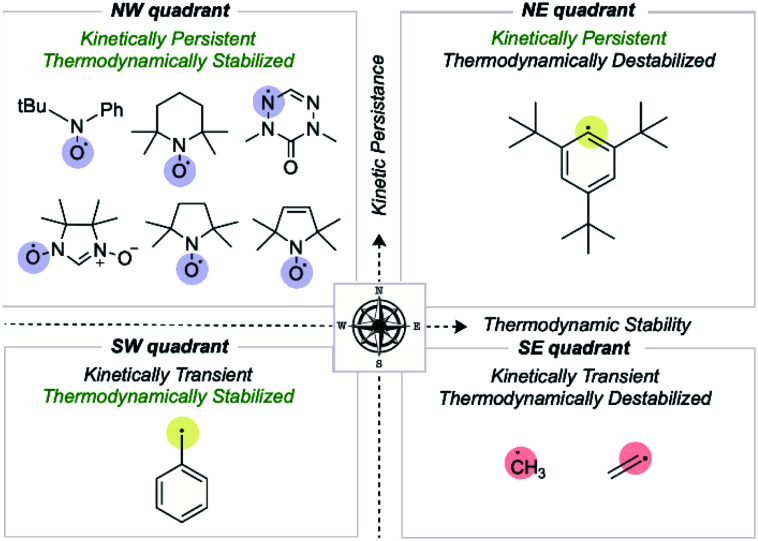
Mapping organic radicals according to two criteria, kinetic persistence and thermodynamic stabilization. The most “stable” organic radicals reside in the NW quadrant.

We first consider how radical structures differ in the two principal characteristics introduced above, kinetic lifetime and thermodynamic stabilization. We can visualize these variations on a map of relative radical stability, which is divided into four quadrants (Fig. 2): (i) thermodynamically destabilized, kinetically transient radicals such as CH_3_˙ in the SE quadrant; (ii) thermodynamically stabilized, kinetically transient structures such as the benzyl radical in the SW quadrant; (iii) thermodynamically destabilized, kinetically persistent radicals such as a sterically protected 1,5-disubstituted phenyl radical in the NE quadrant; (iv) thermodynamically stabilized, kinetically persistent radicals in the NW quadrant. All stable radicals exist in this final quadrant.

The thermodynamic stabilization of radicals can arise from resonance stabilization, such as *via* conjugation with an adjacent π-system or lone-pair, or hyperconjugation with a neighboring σ-bond.^21–25^ Extended conjugation involving several π-bonds allows extensive spin delocalization. For example, the triphenylmethyl radical has an RSE of −24.7 kcal mol^−1^ due to extended conjugation, in contrast to benzyl radical, which is −14.6 kcal mol^−1^.^26^ Another factor influencing radical stability is the element(s) on which the spin density is located. For example, N-, O- and S-centered radicals are disproportionately represented among known stable radical structures.^27^ This observation may appear counterintuitive from a thermodynamic standpoint since the BDE values of X–H bonds are generally larger than C–H bonds. However, these elements possess lone pairs and are highly electronegative, slowing the kinetics of radical dimerization and reaction with molecular O_2_, respectively.^27^ Substituent effects also help stabilize radicals, where the presence of polar groups provides conjugative/inductive effects, which help in charge distribution and spin delocalization. The presence of both donor and acceptor substituents results in appreciable resonance, known as the captodative effect.^28^ Alongside intrinsic structural effects, extrinsic factors such as changes in pH can result in radical stabilization due to changes in orbital configuration that result in the singly occupied molecular orbital (SOMO) no longer being highest in energy.^29^

While thermodynamic stabilization is solely electronic in origin, the kinetics of radical reactions can be profoundly influenced by steric effects. A simple example of an increase in steric bulk resulting in lower reactivity involves replacing H atoms with heavier elements such as Cl.^30^ The incorporation of branched substituents such as *t*-butyl groups close to the radical center have an even more significant effect.^31^ This phenomenon is further exemplified by stable radicals such as (2,2,6,6-tetramethylpiperidin-1-yl)oxyl (TEMPO), where two *gem*-dimethyl groups provide steric protection for the long-lived nitroxyl radical. Sigman and Sanford have illustrated the effects of *N*-alkyl group length in extending the half-life of pyridinium radicals.^32^ Bulky substituents around a radical center result in steric hindrance towards bimolecular reactions, *e.g.*, with other radicals, solvent molecules, and molecular O_2_.

### Towards a quantitative metric for radical stability

We sought to develop numerical descriptors for the two independent dimensions of radical stability: (i) thermodynamic stabilization *via* electron delocalization and (ii) kinetic persistence due to steric hindrance.^33^ In contrast to RSE scales, we were keen to avoid an atom or bond-specific scheme and instead focus on describing the extent of spin-delocalization directly. This led us to consider the largest atomic (Mulliken) spin density as a descriptor for the effect of thermodynamic stabilization. Steric effects on pyridinium radicals' lifetimes have been described previously using multidimensional Sterimol parameters^34,35^ to quantify the out-of-plane distance of *N*-alkyl substituents close to the radical center. To generalize this concept for radicals of arbitrary 3D-geometry, we focused on describing the extent to which adjacent functional groups occupy the space around a radical center. In our work, this is quantified by the percent buried volume, defined as the occupied percent of the total volume of a sphere with a defined radius centered around the radical. Cavallo and Nolan developed the buried volume parameter to capture a coordinated ligand's steric demands around a central metal.^36,37^ To our knowledge, this has not been used previously to describe radicals' kinetic persistence. In our buried volume calculations, we define the radical center as the atom with the maximum fractional spin, which we expect to exert a strong influence on radical reactivity.

**Fig. 3:**
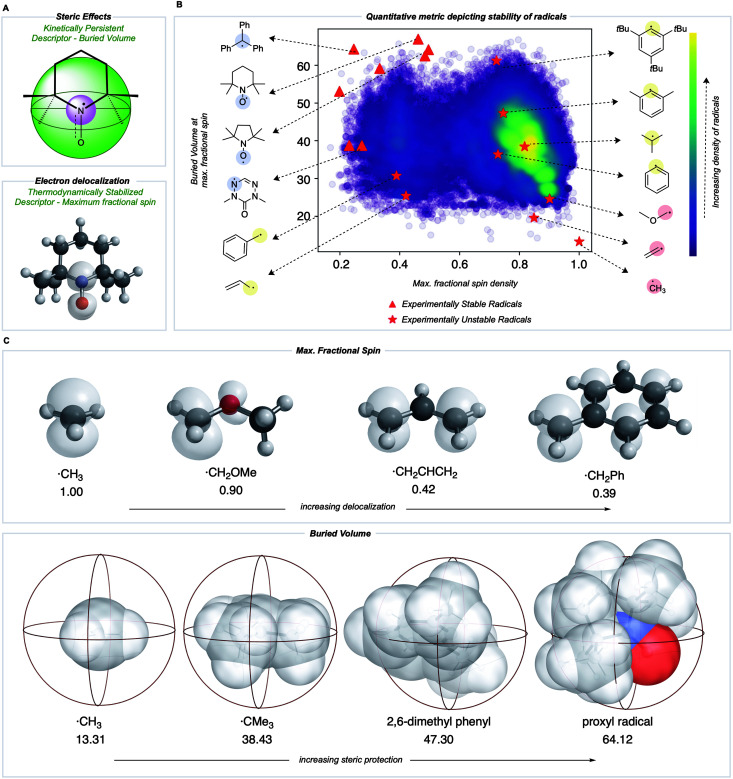
(A) Parameters used to build a stability metric for organic radicals in two dimensions corresponding to kinetic and thermodynamic stabilization. (B) Quantitative evaluation of these metrics produces a map in which experimentally validated stable radicals (red triangle) are separated from common radical structures. (C) Visualization of electronic and steric descriptors.

Most radicals generated in organic chemistry are short-lived and unstable. Therefore, by comparing the spin and buried volumes scores of radicals known experimentally to be stable to a large sample of more typical organic radicals, we can validate that our metric can explain radical stability by separating known-stable from likely unstable radical species. A recently generated database of 200 000 quantum chemical calculations performed at the M06-2X/def2-TZVP^38^ level for small, organic open-shell molecules generated by breaking single, non-cyclic bonds in molecules taken from the PubChem Compound database provides such a sample of expected organic radical configurations.^20,39^ These radicals were obtained after conformer sampling using the MMFF94 force field within RDKit,^40,41^ following which the lowest-energy conformer was utilized for DFT optimizations. Molecules with 10 heavy atoms or fewer containing only C, N, S, O, and H atoms were re-optimized using water as an implicit solvent using Gaussian 16.^42^ All calculations reported in the main text were optimized in water using the SMD solvation model,^43^ while gas-phase values are reported in the ESI (Fig. S1†). Water was used due to its relevance in the performance of redox batteries. For all the radicals in this dataset, Mulliken spin densities were obtained for each atom, and the corresponding buried volume around each atom was computed from the optimized molecular geometry. For each molecule, the computed spin density values were normalized: the absolute magnitudes of heavy atom spins were summed, neglecting the small spin values on H atoms, and then converted to fractional spins that sum to one. The center with the highest fractional spin was assigned as the location of the radical center. Negligible basis set dependence of spin densities was confirmed by comparing with def2QZVP for a small set of radicals (ESI Table S1†). A Python package, DBSTEP, was developed to aid the high-throughput evaluation of buried volumes for almost 90 000 compounds, using numerical integration on a Cartesian grid with a spacing of 0.05 Å. Voxel occupancies were determined based on the unscaled atomic Bondi radii for all atoms. A sphere radius of 3.5 Å was used throughout (Fig. 3A).^44^ The final breakdown of organic radicals in the dataset based on their location of maximum spin density are as follows: 73 080 carbon, 5097 oxygen, 9693 nitrogen, and 1447 sulfur (Fig. S1†).

The distribution of spin density and steric descriptor values obtained for the entire dataset of 73 080 organic radicals is shown in Fig. 3B. We also computed descriptor values for specific radicals whose stability and persistence are experimentally known (red points in Fig. 3B). The appearance of validated stable radicals, such as TEMPO, proxyl, and trityl radicals at the frontier of this distribution (top left corner), was encouraging since these structures score amongst the highest in terms of both thermodynamic stabilization (low spin density) and kinetic persistence (high buried volume). Structures for all plotted stable radical species are depicted in the ESI (Fig. S2†). The benzyl radical appears in the lower-left quadrant, indicative of favorable delocalization but low buried volume around the radical center, consistent with its transience. Resonantly stabilized radicals such as these play an essential role in soot precursor formation, as their relatively long lifetimes and multiple reactive sites lead to further ring growth during combustion. Conversely, the 2,4,6-tri-*tert*-butyl phenyl radical appears in the upper right quadrant due to high buried volume but a highly localized unpaired electron. Finally, small and unstable structures such as the methyl radical appear in the lower right, indicating an absence of either thermodynamic stabilization or kinetic protection. Additionally, a higher density of radicals is obtained in the lower right corner of this map, which signifies that an organic radical chosen at random is likely to be unstable and short-lived.

Both electron delocalization and steric protection play an important role, and some examples of spin density and buried volume descriptors are illustrated in Fig. 3C. The spin density in a methyl radical is fully localized to the carbon atom (our scheme only considers non-hydrogen atoms), giving a value of 1. However, spin delocalization in structures such as the benzyl radical results in a maximum spin density value less than 0.5 on the CH_2_. In the most highly delocalized structures, we obtained maximum spin densities close to 0.2. Further quantitative justification for the use of max. spin density as a descriptor of thermodynamic stability comes from the fact that it is highly correlated with RSE values defined by the cleavage of a particular bond type (*e.g.*, C(sp^3^)–H, *R*^2^ = 0.94) and with the BDE values for over 100 000 C–H bonds (*R*^2^ = 0.66) (see Fig. S5†). Similarly, the buried volume values ranged from 13% for the smallest radicals such as ˙CH_3_ to around 65% for the most sterically protected structures, such as the proxyl radical or TEMPO. These steric descriptor values are most strongly influenced by the degree of substitution at the site of the maximum spin density and of each of the neighboring atoms, as illustrated by the contents of the 3.5 Å radius spheres shown in Fig. 3C. We found the results from using different sphere sizes to generate these buried volumes (2.5, 3.0, 3.5, 4.0 Å) to be very highly correlated (*R*^2^ = 0.96–0.99) and so we have retained the familiar value of 3.5 Å throughout (Fig. S8†).

**Fig. 4:**
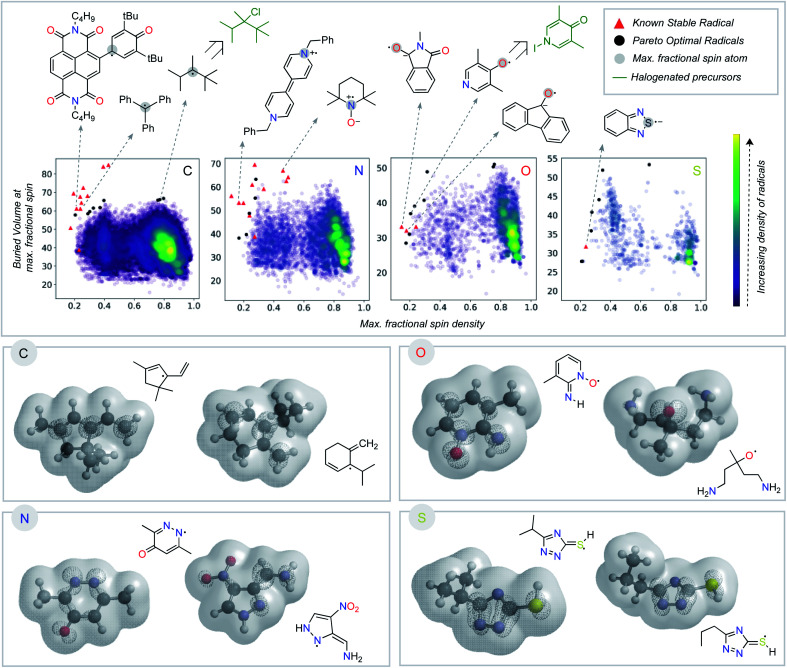
Superimposition of known stable radicals with optimal predicted organic radicals according to fractional spin density and buried volume parameters. Analysis separated into C, N, O, and S- radical centers. Below, spin densities and molecular isosurfaces are show for selected molecules from the Pareto front.

Having seen that empirically validated stable radicals occupy a distinct region of our parameter space, we next focused on identifying other molecules predicted to be similarly stable. This analysis relies on identifying the Pareto optimal set of radicals – those structures for which there are no other examples superior both in terms of buried volume and delocalization (Fig. 4).^45,46^ We added several charged structures known to be stable radicals (*e.g.*, phenyl-viologen) to our dataset (ESI Fig. S2†) at this point. The Pareto frontier set of radicals was identified from our large dataset, separated according to the atom (C, N, O, or S) with the largest spin density. The proximity of some of these computationally derived structures to known stable radicals (red triangles) encouraged us to explore these as potential new stable radical candidates.

Some of the computationally optimized molecules on the Pareto frontier are shown in Fig. 4. They are divided into the type of atom the radical center lies on upon DFT optimization. All structures contain high delocalization, steric protection, or both. Based on their shared features with currently known stable radicals, we propose that compounds on the Pareto frontier are potential candidates for long-lived radicals. As an illustration, two such radicals computationally predicted to be Pareto optimal could be synthesized in one step from commercially available halogenated precursors (shown in green, Fig. 4).^47–49^ Our newly developed metric is, therefore, able to aid in the identification of stable radical structures. On a general note, stable radicals lie on the top left region of each graph based on atom type. However, we also observe that stable heteroatom radicals tolerate lower buried volumes (as low as 40%) than their carbon-centered counterparts. As discussed above, we attribute this to their electronegativity and lone-pairs, which retard reactions at the radical center.

### Comparison to radical stabilization energies

RSE values are commonly invoked to quantify radical stability.^10,11^ In this section, we compare our newly developed stability metric with traditional RSE values of carbon-centered radicals, derived from the difference in C–H BDE values relative to methane. To do this, we formulated our two parameters into a singular stability metric (eqn (1)).

1 Radical stability score = *V*_bur_ + 50 × (1 − max. spin)

**Fig. 5:**
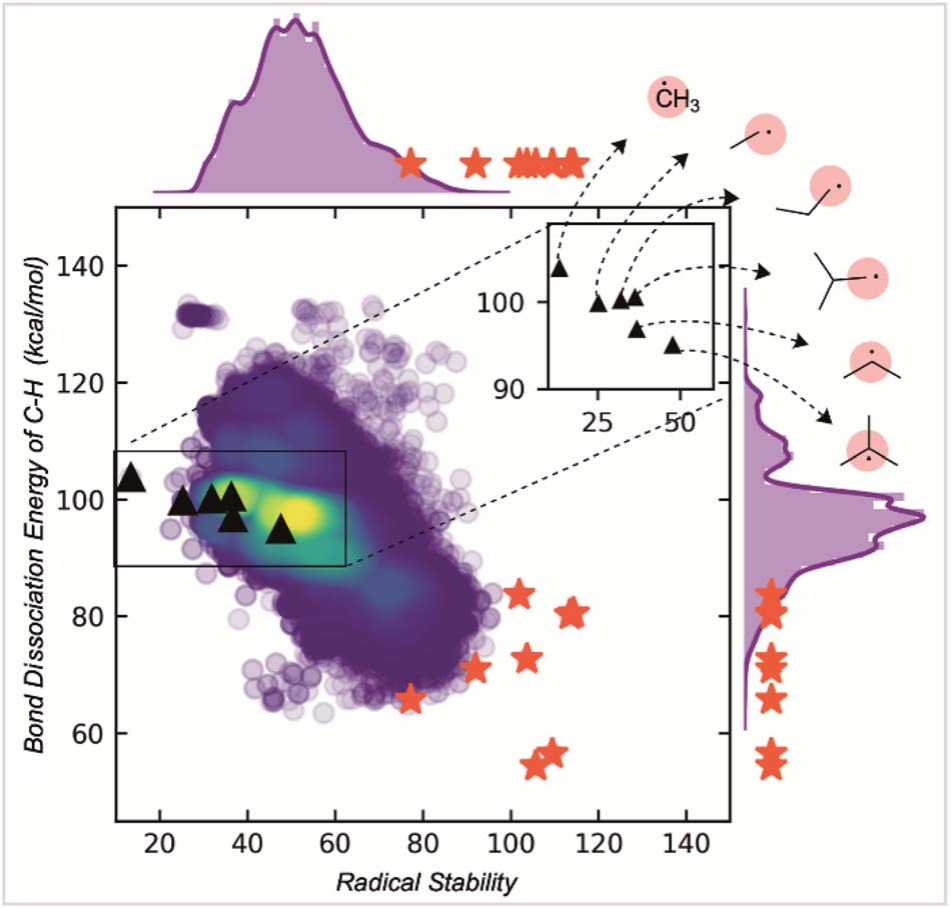
Correlation of radical stability score with C–H BDE. The red stars are known stable radicals.

The factor of 50 in this equation was chosen to give approximately equal weight to buried volume and spin terms, reflecting kinetic and thermodynamic contributions to radical stability. In contrast, RSE or BDE values are purely thermodynamic. The M06-2X/def2-TZVP gas-phase BDE values for 107 717 C–H bonds were collected for our dataset of 200 000 open-shell molecules. Comparing the number of C–H bonds to the C-centered radicals (73 080), the increase is due to the fragmentation scheme from a parent molecule. Multiple parent molecules can generate the same C-centered radical, corresponding to different C–H bonds breaking reactions. We compare these BDE values with our Radical Stability Score (RSS) in Fig. 5.

While RSS values are inversely correlated with BDEs, the correlation is relatively modest (*R*^2^ = 0.6). The RSS values provide different information, as illustrated for a family of alkyl radicals (Fig. 5). Relative C–H BDE values demonstrate the sequence of thermodynamic stabilities: methyl < primary < secondary < tertiary radicals. However, on solely thermodynamic grounds, there is little to distinguish between the stabilities of primary ethyl, *n*-propyl and i-propyl radicals, and the *t*-butyl radical is only marginally more stable than the secondary i-propyl radical. RSS values preserve the overall order of stabilities of primary, secondary, and tertiary radicals. However, they also provide additional stratification: the primary radicals are separated with longer or bulkier alkyl chains contributing to a greater stability score. The tertiary radical is more clearly differentiated from the rest of the structures due to steric effects. We suggest that this scale offers greater resolution to separate stable from unstable candidates. Furthermore, the RSS score exhibits a good correlation (*R*^2^ = 0.85) with the relative rates of decomposition of 18 radicals, which exhibit second order kinetics consistent with radical homocoupling (Fig. S9†).

RSS values are also more helpful than BDE values for classification tasks. For example, the extreme instability of the methyl radical can be inferred from the fact that it is a statistical outlier in the overall distribution of RSS values. This is not the case when looking at the BDE distribution (ESI Fig. S6†). At the other and potentially more helpful end of the spectrum, empirically stable radicals are also clustered towards the top end of the RSS distribution (red stars, Fig. 5), more so than for the distribution of BDE values, which were obtained using a machine learning BDE prediction tool, ALFABET.^39^ All known stable radicals considered in this work are found in or above the 97th percentile of RSS values, which drops slightly to the 93rd percentile for BDE value. Therefore, we anticipate that the use of RSS values in predictive screening for new stable radicals will result in greater enrichment compared to more traditional metrics.

### Utility in studies of radical cascade reactions

**Fig. 6:**
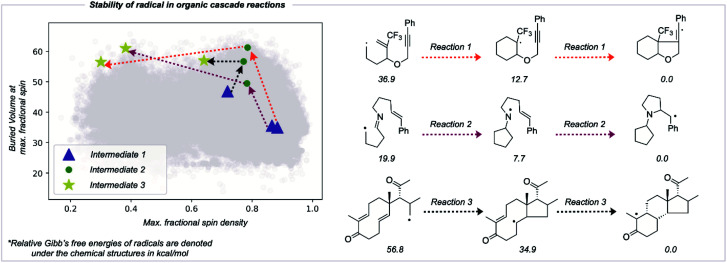
Improvement in radical stability in consecutive intermediates in organic radical cascade reactions. The type of arrows on the reaction depicts the respective reaction on the stability graph. Computationally optimized molecules are depicted in grey in the background.

The RSS metric and the underlying two descriptions of thermodynamic stabilization and kinetic persistence enable radicals' stabilities to be compared quantitatively. We assessed the utility of this approach in comparing radical intermediates occurring sequentially along a reaction pathway as a collective variable for the reaction coordinate. We studied three cascade reactions involving sequential radical *exo*-trig/dig cyclization steps,^50–53^ computing the thermochemistry at the M062X/def2TZVP level of theory (Fig. 6). Each successive intermediate is more stable than the last, and while this is influenced by the nature of bonds formed and broken, the evolution of the fractional spin descriptor illustrates the contribution from greater radical delocalization being particularly noticeable in the second step, while the main increase in kinetic protection occurs in the first step. The final values of two of these structures suggest that these would be somewhat stable radicals with appreciable lifetimes. While the RSS metric describes structural and electronic changes around the site of an unpaired electron, other contributions to a reaction's driving force, such as strain-release from ring-opening, may also play a key role and would need to be separately accounted for.^54^

## Conclusion

Inspired by thermodynamic and kinetic considerations, we propose two key computational descriptors for quantifying radical stability, the maximum spin density and the buried volume around the atom where this spin is located. Two-dimensional plots of these descriptors were generated for thousands of common radicals and for experimentally verified stable, highly persistent radicals. Stable radicals appear in a distinct region of this feature space associated with thermodynamic stability and kinetic persistence. This analysis gives rise to the notion that stable organic radicals can be computationally predicted and designed in a quantitative fashion. The two dimensions of radical stability can be considered independently or combined into a Radical Stability Score (RSS). This metric preserves the thermodynamic information contained in traditional metrics derived from BDE values while also allowing for differentiation between sterically distinct radical centers. Overall, this enables more straightforward distinctions between the small population of stable radicals and the vast majority of shorter-lived structures. We also demonstrated the use of these descriptors to compare successive radical intermediates occurring along a reaction pathway. This work provides a unified evaluation of radical stability that could be incorporated as an objective function in molecular design efforts using high-throughput screening or generative machine learning. The discovery of new, more stable radicals for use in electronic devices such as redox batteries, transistors, and light-emitting diodes is our overarching goal.

## Data availability

The datasets generated and analyzed during the current study are available on Figshare with the identifier DOI: 10.6084/m9.figshare.14597556.v2. All data for the fractional spin and buried volumes has been provided in a CSV file for the water-phase calculations of the computationally optimized molecules. Cartesian coordinates of the optimized molecules are included in the SDF file format. In the spin and buried volume CSV, atom_index corresponds to the canonical atom order assigned by RDKit and is also represented by the atom ordering for the corresponding entry in the SDF file. Similar data for experimentally-known species is provided in the ESI Section 4.† The data corresponding to the organic cascade reactions are provided in the ESI Section 9.†

## Author contributions

PSJ and RSP conceptualized this work; SSSV and PSJ performed quantum chemical calculations, curated quantum chemical data and molecular features. All authors participated in formal analysis, manuscript preparation, revisions, and editing.

## Conflicts of interest

There are no conflicts to declare.

## Supplementary Material

SC-012-D1SC02770K-s001

## References

[cit1] Tang B., Zhao J., Xu J.-F., Zhang X. (2020). Tuning the stability of organic radicals: from covalent approaches to non-covalent approaches. Chem. Sci..

[cit2] Gomberg M. (1900). An instance of trivalent carbon: triphenylmethyl. J. Am. Chem. Soc..

[cit3] Griller D., Ingold K. U. (1976). Persistent carbon-centered radicals. Acc. Chem. Res..

[cit4] Wilcox D. A., Agarkar V., Mukherjee S., Boudouris B. W. (2018). Stable Radical Materials for Energy Applications. Annu. Rev. Chem. Biomol. Eng..

[cit5] Gracia R., Mecerreyes D. (2013). Polymers with redox properties: materials for batteries, biosensors and more. Polym. Chem..

[cit6] Root S. E., Savagatrup S., Printz A. D., Rodriquez D., Lipomi D. J. (2017). Mechanical Properties of Organic Semiconductors for Stretchable, Highly Flexible, and Mechanically Robust Electronics. Chem. Rev..

[cit7] Boudouris B. (2013). Engineering optoelectronically active macromolecules for polymer-based photovoltaic and thermoelectric devices. Curr. Opin. Chem. Eng..

[cit8] Ji L., Shi J., Wei J., Yu T., Huang W. (2020). Air-Stable Organic Radicals: New-Generation Materials for Flexible Electronics?. Adv. Mater..

[cit9] Berville M., Richard J., Stolar M., Choua S., Le Breton N., Gourlaouen C., Boudon C., Ruhlmann L., Baumgartner T., Wytko J. A., Weiss J. (2018). A Highly Stable Organic Radical Cation. Org. Lett..

[cit10] Wood G. P. F., Moran D., Jacob R., Radom L. (2005). Bond Dissociation Energies and Radical Stabilization Energies Associated with Model Peptide-Backbone Radicals. J. Phys. Chem. A.

[cit11] Henry D. J., Parkinson C. J., Mayer P. M., Radom L. (2001). Bond Dissociation Energies and Radical Stabilization Energies Associated with Substituted Methyl Radicals. J. Phys. Chem. A.

[cit12] Luo Y.-R., Comprehensive Handbook of Chemical Bond Energies, 2007, pp. 1–1656

[cit13] Wodrich M. D., Mckee W. C., Schleyer P. V. R. (2011). On the Advantages of Hydrocarbon Radical Stabilization Energies Based on R–H Bond Dissociation Energies. J. Org. Chem..

[cit14] Coote M. L., Lin C. Y., Zavitsas A. A. (2014). Inherent and transferable stabilization energies of carbon- and heteroatom-centred radicals on the same relative scale and their applications. Phys. Chem. Chem. Phys..

[cit15] Hioe J., Šakić D., Vrček V., Zipse H. (2015). The stability of nitrogen-centered radicals. Org. Biomol. Chem..

[cit16] Zipse H., Radical Stability—A Theoretical Perspective, in Radicals in Synthesis I, Springer-Verlag, 2006, pp. 163–189

[cit17] Hioe J. and Zipse H., Encyclopedia of Radical in Chemistry, Biology and Materials, John Wiley & Sons Ltd, Chichester, UK, 2012, pp. 449–477

[cit18] Coote M. L., Lin C. Y. and Zipse H., Carbon-Centered Free Radicals and Radicals Cations, John Wiley & Sons, 2010, pp. 83–10

[cit19] Zavitsas A. A. (2008). A Single Universal Scale of Radical Stabilization Energies Does Not Exist: Global Bond Dissociation Energies and Radical Thermochemistries Are Described by Combining Two Universal Scales. J. Org. Chem..

[cit20] St. John P. C., Guan Y., Kim Y., Etz B. D., Kim S., Paton R. S. (2020). Quantum chemical calculations for over 200,000 organic radical species and 40,000 associated closed-shell molecules. Sci. Data.

[cit21] Gobbi A., Frenking G. (1994). Resonance Stabilization in Allyl Cation, Radical, and Anion. J. Am. Chem. Soc..

[cit22] Kossiakoff A., Rice F. O. (1943). Thermal Decomposition of Hydrocarbons, Resonance Stabilization and Isomerization of Free Radicals. J. Am. Chem. Soc..

[cit23] Bader R. F. W., Slee T. S., Cremer D., Kraka E. (1983). Description of conjugation and hyperconjugation in terms of electron distributions. J. Am. Chem. Soc..

[cit24] Alabugin I. V., Dos Passos Gomes G., Abdo M. A. (2019). Hyperconjugation. Wiley Interdiscip. Rev.: Comput. Mol. Sci..

[cit25] Mulliken R. S., Rieke C. A., Brown W. G. (1941). Hyperconjugation*. J. Am. Chem. Soc..

[cit26] Kubo T. (2019). Synthesis, Physical Properties, and Reactivity of Stable, π-Conjugated, Carbon-Centered Radicals. Molecules.

[cit27] Hicks R. G. (2007). What's new in stable radical chemistry?. Org. Biomol. Chem..

[cit28] Viehe H. G., Janousek Z., Merenyi R., Stella L. (1985). The captodative effect. Acc. Chem. Res..

[cit29] Gryn'Ova G., Marshall D. L., Blanksby S. J., Coote M. L. (2013). Switching radical stability by pH-induced orbital conversion. Nat. Chem..

[cit30] Veciana J., Carilla J., Miravitlles C., Molins E. (1987). Free radicals as clathrate hosts: crystal and molecular structure of 1: 1 perchlorotriphenylmethyl radical–benzene. J. Chem. Soc., Chem. Commun..

[cit31] Degirmenci I., Coote M. L. (2016). Effect of Substituents on the Stability of Sulfur-Centered Radicals. J. Phys. Chem. A.

[cit32] Sevov C. S., Hickey D. P., Cook M. E., Robinson S. G., Barnett S., Minteer S. D., Sigman M. S., Sanford M. S. (2017). Physical Organic Approach to Persistent, Cyclable, Low-Potential Electrolytes for Flow Battery Applications. J. Am. Chem. Soc..

[cit33] Gallegos L. C., Luchini G., St. John P. C., Kim S., Paton R. S. (2021). Importance of Engineered and Learned Molecular Representations in Predicting Organic Reactivity, Selectivity, and Chemical Properties. Acc. Chem. Res..

[cit34] Verloop A., Drug Design, Academic Press, New York, 1976, Vol. III

[cit35] Brethomé A. V., Fletcher S. P., Paton R. S. (2019). Conformational Effects on Physical-Organic Descriptors: The Case of Sterimol Steric Parameters. ACS Catal..

[cit36] Hillier A. C., Sommer W. J., Yong B. S., Petersen J. L., Cavallo L., Nolan S. P. (2003). A Combined Experimental and Theoretical Study Examining the Binding ofN-Heterocyclic Carbenes (NHC) to the Cp*RuCl (Cp* = η5-C5Me5) Moiety: Insight into Stereoelectronic Differences between Unsaturated and Saturated NHC Ligands. Organometallics.

[cit37] Falivene L., Credendino R., Poater A., Petta A., Serra L., Oliva R., Scarano V., Cavallo L. (2016). SambVca 2. A Web Tool for Analyzing Catalytic Pockets with Topographic Steric Maps. Organometallics.

[cit38] Zhao Y., Truhlar D. G. (2008). The M06 suite of density functionals for main group thermochemistry, thermochemical kinetics, noncovalent interactions, excited states, and transition elements: two new functionals and systematic testing of four M06-class functionals and 12 other function. Theor. Chem. Acc..

[cit39] St. John P. C., Guan Y., Kim Y., Kim S., Paton R. S. (2020). Prediction of organic homolytic bond dissociation enthalpies at near chemical
accuracy with sub-second computational cost. Nat. Commun..

[cit40] Landrum G., RDKit: Open-Source Cheminformatics Software, 2016, http://www.rdkit.org/, https://github.com/rdkit/rdkit

[cit41] Riniker S., Landrum G. A. (2015). Better Informed Distance Geometry: Using What We Know To Improve Conformation Generation. J. Chem. Inf. Model..

[cit42] Frisch M. J., Trucks G. W., Schlegel H. B., Scuseria G. E., Robb M. A., Cheeseman J. R., Scalmani G., Barone V., Petersson G. A., Nakatsuji H., Li X., Caricato M., Marenich A. V., Bloino J., Janesko B. G., Gomperts R., Mennucci B., Hratchian H. P., Ortiz J. V., Izmaylov A. F., Sonnenberg J. L., Williams-Young D., Ding F., Lipparini F., Egidi F., Goings J., Peng B., Petrone A., Henderson T., Ranasinghe D., Zakrzewski V. G., Gao J., Rega N., Zheng G., Liang W., Hada M., Ehara M., Toyota K., Fukuda R., Hasegawa J., Ishida M., Nakajima T., Honda Y., Kitao O., Nakai H., Vreven T., Throssell K., Montgomery Jr J. A., Peralta J. E., Ogliaro F., Bearpark M. J., Heyd J. J., Brothers E. N., Kudin K. N., Staroverov V. N., Keith T. A., Kobayashi R., Normand J., Raghavachari K., Rendell A. P., Burant J. C., Iyengar S. S., Tomasi J., Cossi M., Millam J. M., Klene M., Adamo C., Cammi R., Ochterski J. W., Martin R. L., Morokuma K., Farkas O., Foresman J. B. and Fox D. J., Gaussian 16 Rev. C.01, Wallingford, CT, 2016

[cit43] Marenich A. V., Cramer C. J., Truhlar D. G. (2009). Universal Solvation Model Based on Solute Electron Density and on a Continuum Model of the Solvent Defined by the Bulk Dielectric Constant and Atomic Surface Tensions. J. Phys. Chem. B.

[cit44] Luchini G. and Paton R. S., DBSTEP: DFT Based Steric Parameters, 2021, Zenodo, 10.5281/zenodo.4702097, https://www.github.com/patonlab/DBSTEP

[cit45] Brethomé A. V., Paton R. S., Fletcher S. P. (2019). Retooling Asymmetric Conjugate Additions for Sterically Demanding Substrates with an Iterative Data-Driven Approach. ACS Catal..

[cit46] Janet J. P., Ramesh S., Duan C., Kulik H. J. (2020). Accurate Multiobjective Design in a Space of Millions of Transition Metal Complexes with Neural-Network-Driven Efficient Global Optimization. ACS Cent. Sci..

[cit47] Baxter A., Brown J. A., Hirs D., Humphreys P., Jones K. L. and Patel V. K., Imidazole derivatives and their use in the treatment of autoimmune or inflammatory diseases or cancers, US Pat., US20190175571, 2019

[cit48] Yamauchi N., Kubota T. and Nyugaku T., Alkoxysilane composition, Jp. Pat., JP-2015212338-A, 2015

[cit49] Lalevee J. and Fouassier J. P., Encyclopedia of Radical in Chemistry, Biology and Materials, John Wiley & Sons, Weinheim, 2012, vol. 1, pp. 37–57

[cit50] McCarroll A. J., Walton J. C. (2001). Programming Organic Molecules: Design and Management of Organic Syntheses through Free-Radical Cascade Processes. Angew. Chem..

[cit51] Tomida S., Doi T., Takahashi T. (1999). New approach to the progesterone BCD-ring system by utilizing a tandem transannular radical cyclization. Tetrahedron Lett..

[cit52] Morikawa T., Nishiwaki T., Kobayashi Y. (1989). Radical cyclization to the trifluoromethyl-substituted double bond: Regioselectivity and tandem cyclization. Tetrahedron Lett..

[cit53] Bowman W. R., Stephenson P. T., Young A. R. (1995). Synthesis of nitrogen heterocycles using tandem radical cyclisation of imines. Tetrahedron Lett..

[cit54] Beckwith A. L. J., Bowry V. W. (1989). Kinetics and regioselectivity of ring opening of substituted cyclopropylmethyl radicals. J. Org. Chem..

