# Supplementary material for: A quantitative metric for organic radical stability and persistence using thermodynamic and kinetic features
Source: Chem Sci. 2021 Sep 6;12(39):13158–66. doi: 10.1039/d1sc02770k (PMC8514092; doi:10.1039/d1sc02770k)
Supplement: SC-012-D1SC02770K-s001 [file SC-012-D1SC02770K-s001.pdf]

Electronic Supplementary Material (ESI) for Chemical Science.
This journal is © The Royal Society of Chemistry 2021

Supplementary Materials for

A Quantitative Metric for Organic Radical Stability and Persistence Using Thermodynamic and Kinetic Features

Shree Sowndarya S.V[†], Peter St. John[‡*], Robert S. Paton[†*]

[†]Department of Chemistry, Colorado State University, Fort Collins, CO, 80523, USA
[‡]Biosciences Center, National Renewable Energy Laboratory, Golden, CO 80401, USA
Corresponding e-mail: peter.stjohn@nrel.gov; robert.paton@colostate.edu

TABLE OF CONTENTS

Section	Description	Page No.
1.	Dependence of basis set for spin density calculations.	S2
2.	Distribution of fractional spin and buried volume of data set.	S3
3.	Structures considered for evaluating the organic radical stability metric.	S4
4.	Fractional spin and buried volumes for experimentally known radicals.	S5
5.	Plot of fractional spin vs. buried volume for radicals optimized in the gas phase.	S6
6.	Plot of pareto-front for radicals optimized in gas phase.	S7
7.	Comparison of Max fractional spin with thermodynamic quantities.	S8
8.	IQR plots for Radical Stability and Radical stabilization energies.	S9
9.	Structures of stable radicals for the comparison of C-H BDE values.	S10
10.	Fractional spins, buried volumes and thermochemistry of radical cascade reactions.	S11
11.	Correlation of RSS metric and relative bimolecular rates of radical decomposition.	S12
12.	RSS metrics generated with different radii used for buried volume analysis.	S13

1. Dependence of basis set for spin density calculations

Table S1. Largest Fractional Spin Density with M06-2X using two basis sets: def2TZVP and def2QZVP. There is small basis set dependence (0.01-0.03) for the value of the normalized fractional spin.

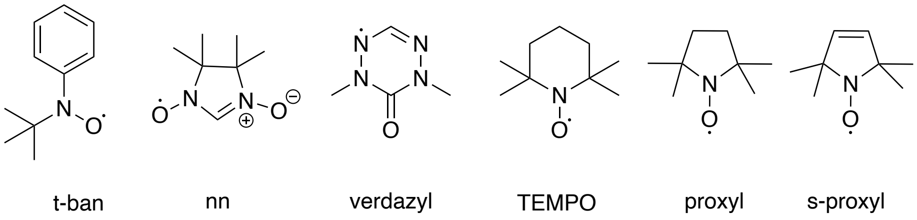

Name	def2-TZVP		def2-QZVP	
	spin density	fractional spin	spin density	fractional spin
nn	0.31	0.20	-0.37	0.21
proxyl	0.54	0.50	0.49	0.47
s-proxyl	0.53	0.48	0.49	0.46
TBAN	0.48	0.33	0.46	0.31
TEMPO	0.52	0.46	0.48	0.45
verdazyl	0.41	0.27	0.39	0.25

2. Distribution of fractional spin and buried volume of data set

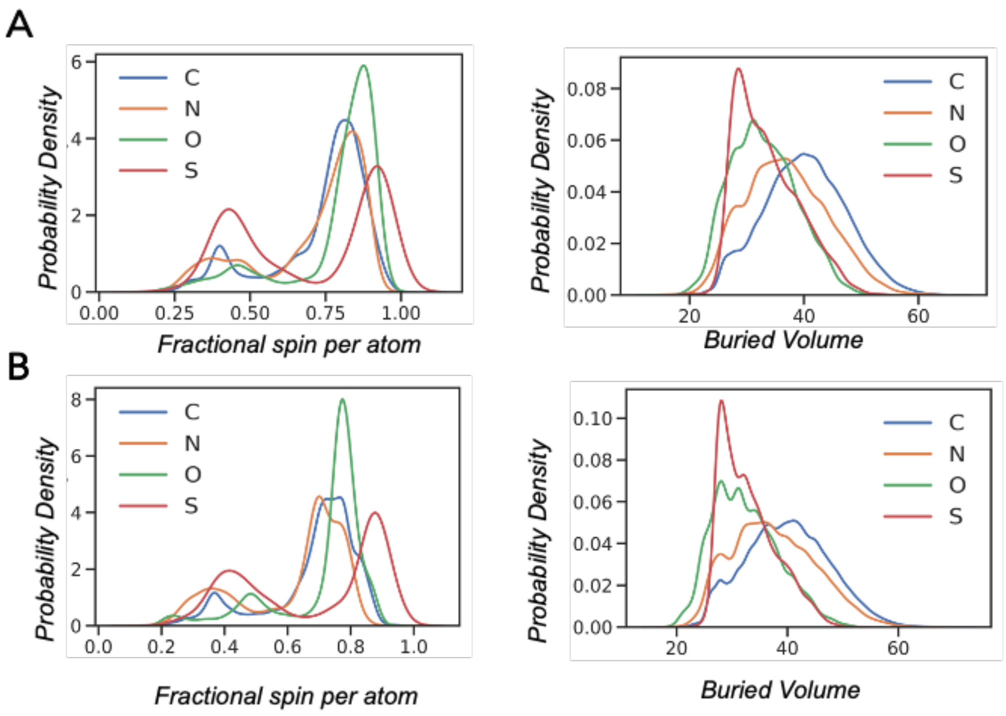

Fig. S1. The kernel density plots represent the spread of buried volume and fractional spin based on atom type in the data set. (A) in water phase (B) in gas phase

3. Structures considered for evaluating the organic radical stability metric

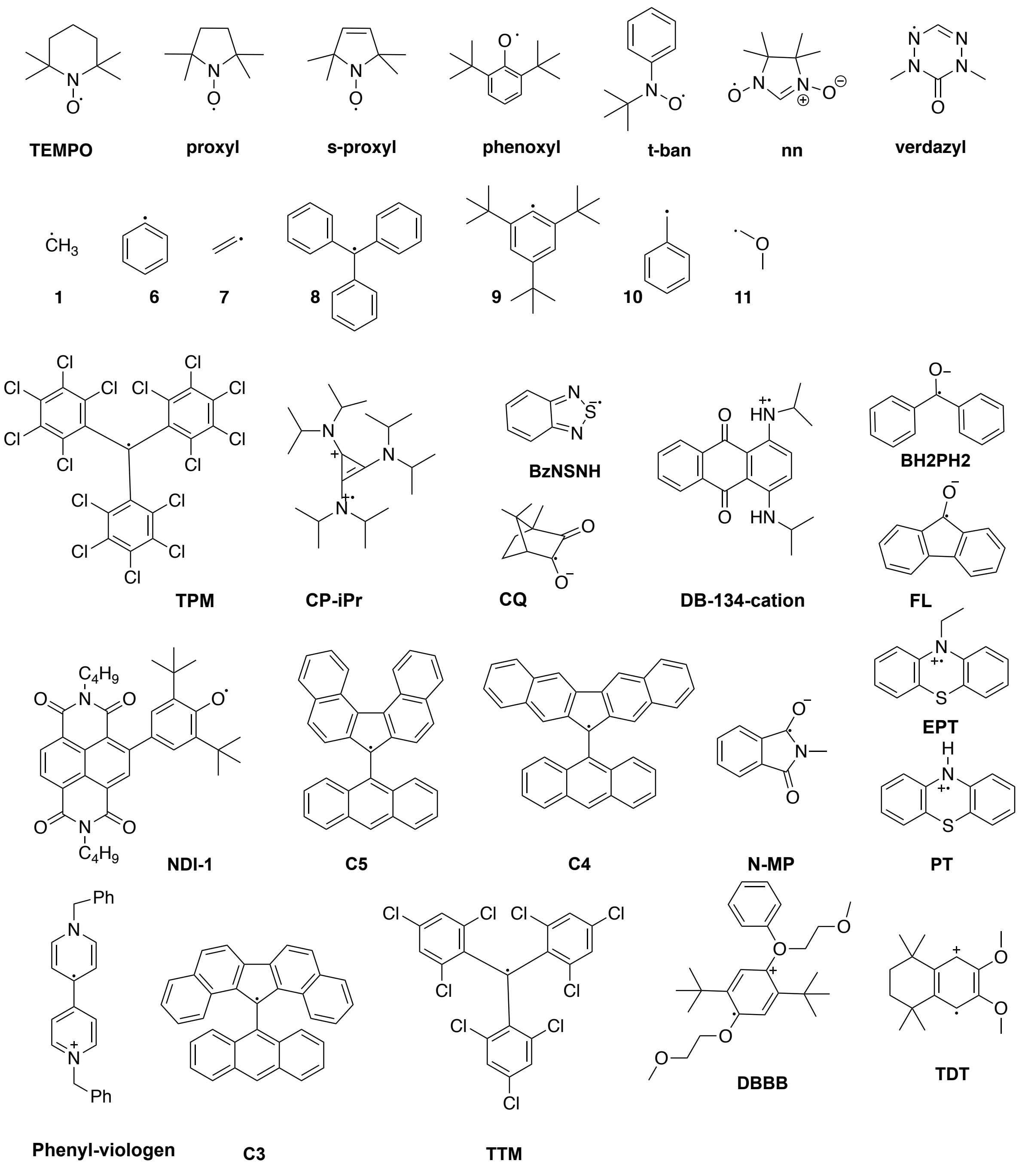

Fig. S2. Structures of radicals considered in the study of organic radical stability.

4. Fractional spin and buried volumes for experimentally known radicals

Table S2. M062X/def2-TZVP fractional Spin Density and V_{bur} (%) for selected stable radicals in gas and water phase.

	Water			*Gas phase*		
**Radical**	**Atom**	**V_{bur} (%)**	**Max. spin**	**Atom**	**V_{bur} (%)**	**Max. spin**
**TPM**	C	84.83	0.4275	C	84.80	0.4271
**CP-iPr**	N	69.47	0.2731	N	69.41	0.2569
**Proxyl**	N	64.13	0.4954	O	43.95	0.5037
**CQ**	O	33.12	0.2407	O	32.98	0.2922
**TEMPO**	N	66.92	0.4611	O	46.85	0.4865
**NDI-1**	C	61.13	0.2139	C	60.93	0.1999
**s-proxyl**	N	62.45	0.4840	O	43.52	0.4898
**car-C5**	C	67.99	0.2872	C	67.97	0.2840
**verdazyl**	N	38.75	0.2730	N	38.75	0.2808
**car-C4**	C	67.62	0.2384	C	67.62	0.2366
**Phenoxyl**	C	38.76	0.2306	C	38.70	0.2149
**phenyl-viologen**	N	53.19	0.1680	N	53.11	0.1667
**car-C3**	C	72.45	0.2585	C	72.51	0.2622
**Nn**	N	53.12	0.1984	O	36.12	0.2254
**TTM**	C	83.93	0.3929	C	83.89	0.3959
**DBBB**	C	61.05	0.2391	C	60.89	0.2236
**TDT**	C	69.35	0.1941	C	69.30	0.2251
**Tban**	N	59.13	0.3330	O	41.54	0.3491
**BP**	C	50.61	0.1736	O	36.47	0.1819
**Trityl (8)**	C	64.27	0.2460	C	64.31	0.2457
**N-MP**	O	33.09	0.1459	C	52.62	0.1595
**FL**	O	31.99	0.1778	O	32.08	0.2438
**DB-134-cation**	N	56.14	0.1181	N	56.13	0.1347
**EPT**	N	60.93	0.2565	N	60.92	0.2413
**PT**	N	47.83	0.2388	S	43.85	0.2460
**BzNSN**	S	31.68	0.2364	S	31.69	0.2098
**11**	C	24.61	0.9008	C	24.58	0.8963
**1**	C	13.32	1.0000	C	13.30	1.0000
**10**	C	30.77	0.3886	C	30.75	0.3914
**7**	C	19.63	0.8487	C	19.61	0.8501
**6**	C	36.39	0.7280	C	36.36	0.7317
**9**	C	61.18	0.7230	C	61.30	0.7249

5. Plot of fractional spin *vs.* buried volume for radicals optimized in the gas phase

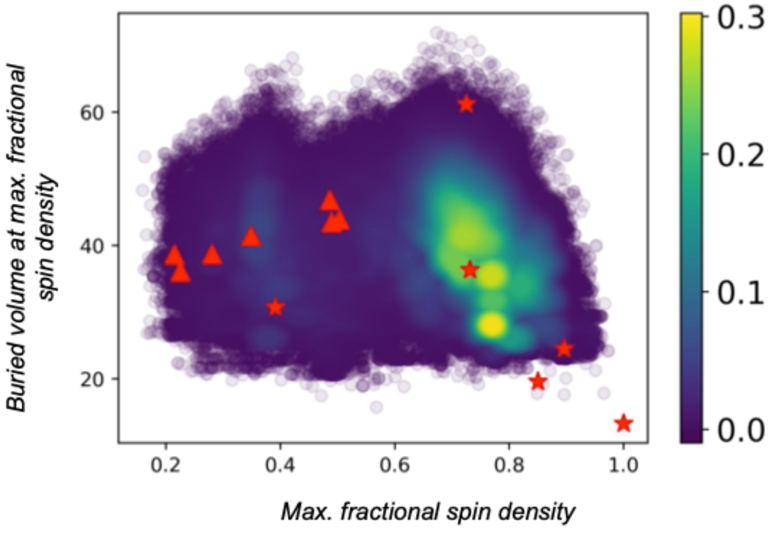

Fig. S3. Depiction of the stability metric to classify know radicals according to their stability. Experimentally stable radicals are shown as red triangles, located in the top left region of the graph.

6. Plot of pareto-front for radicals optimized in gas phase

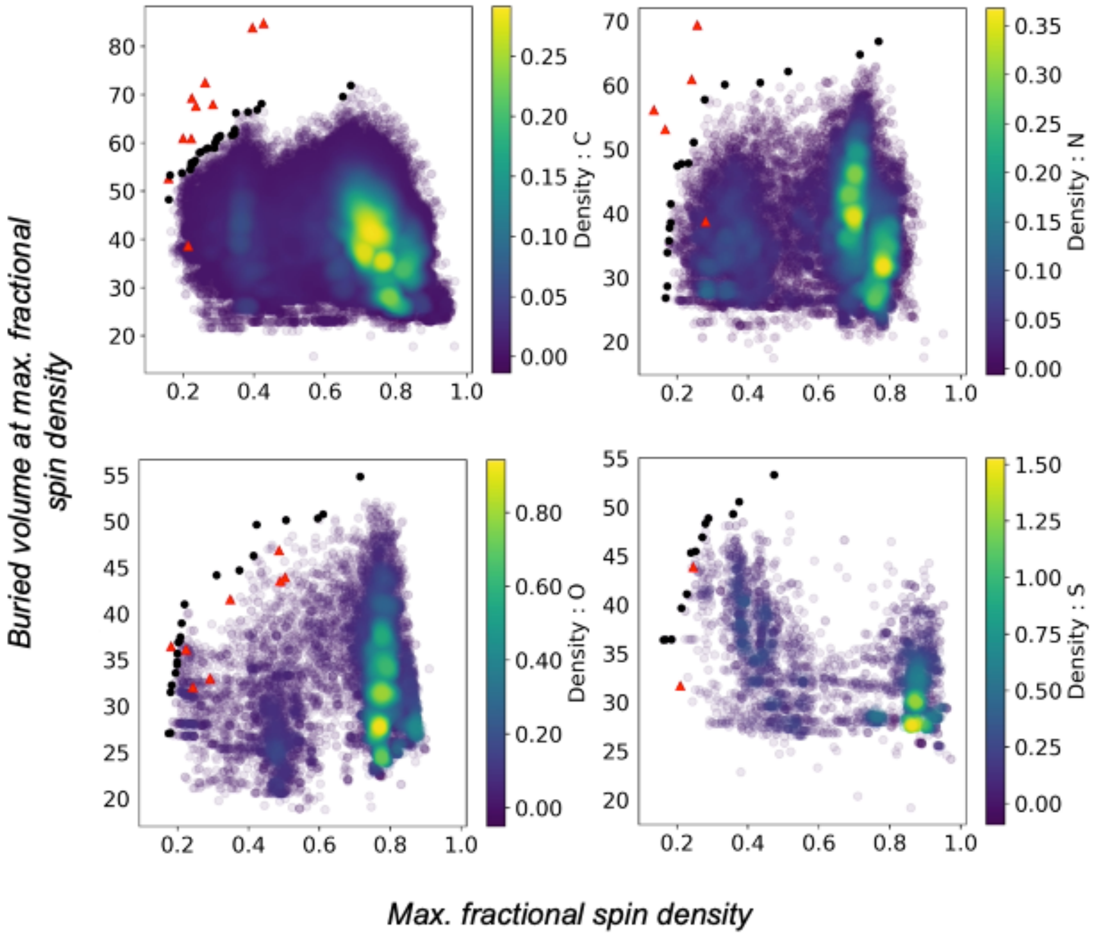

Fig. S4. Pareto front plots based on atom type (C, N, O, S) using fractional spin density and buried volume parameters.

7. Comparison of Max fractional spin with thermodynamic quantities.

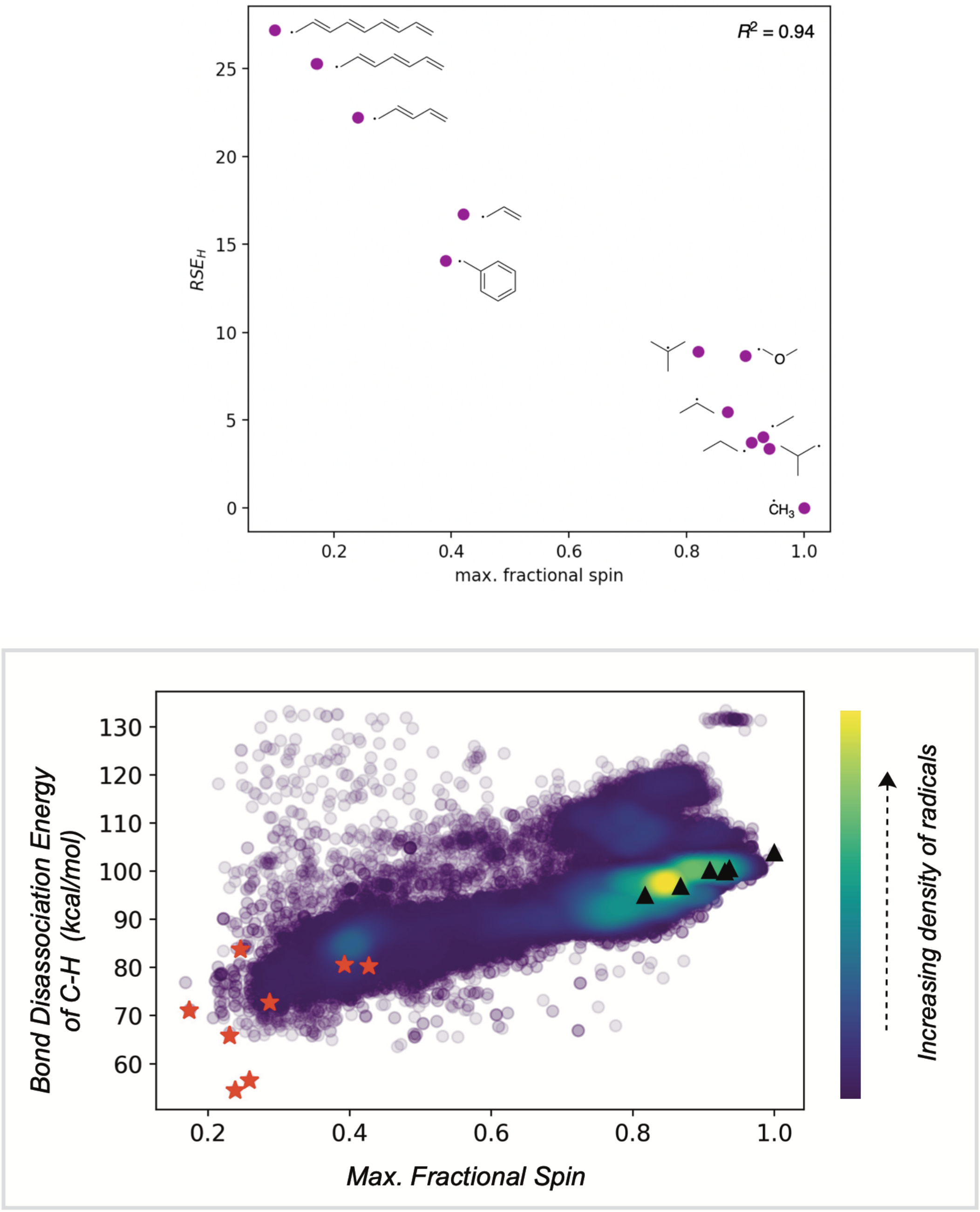

Fig. S5. Top: Correlation of max. fractional spin with RSE involving the cleavage of a C(sp^3)-H bond. Bottom: Correlation of max. fractional spin with C–H BDE. The red stars are known stable radicals. The black triangles are aliphatic hydrocarbons

8. IQR plots for Radical Stability and Radical stabilization energies.

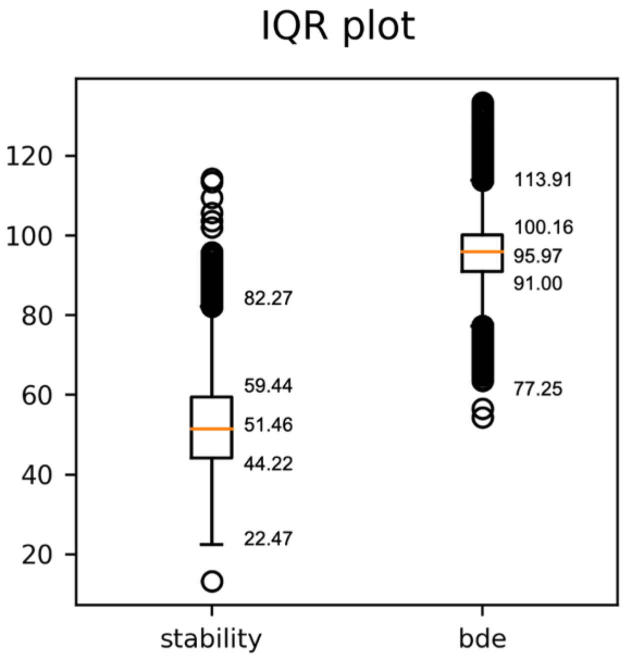

Fig. S6. Chemical molecular structures of stable radicals considered for comparison in C-H bond disassociation energies with the radical stability metric.

9. Structures of stable radicals for the comparison of C-H BDE values.

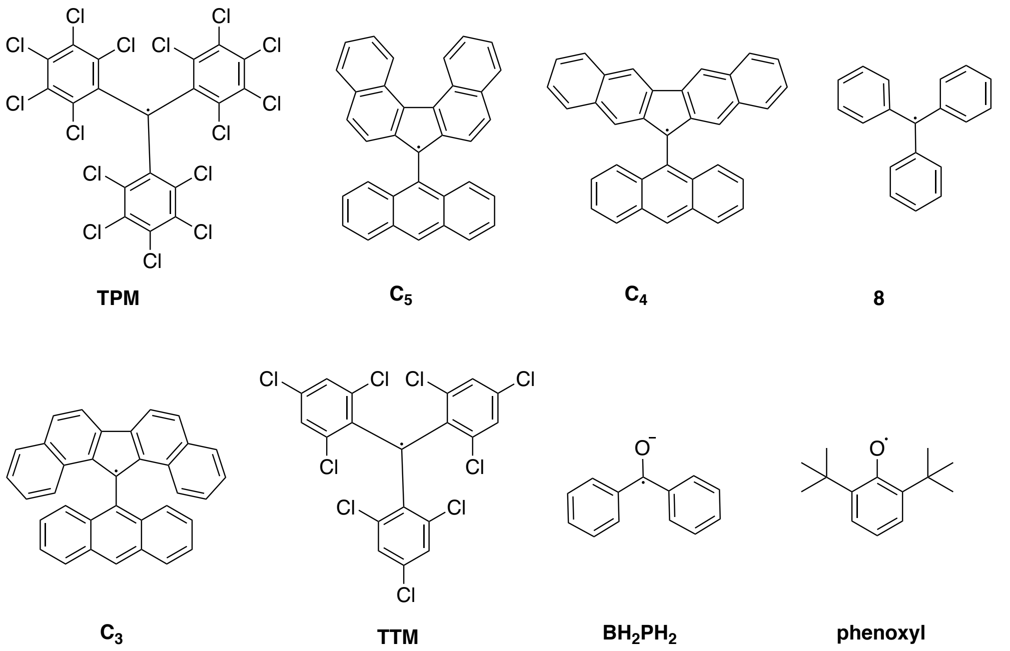

Fig. S7. Molecular structures of stable radicals for which C-H bond dissociation energies are compared with the radical stability (RSS) metric.

10. Fractional spins, buried volumes and thermochemistry of radical cascade reactions.

Table S3. Fractional Spin Density and Buried Volume for the Organic Reaction Cascades.

Rxn	Int	Fractional Spin	Buried Volume	E (Hartree)	ZPE (Hartree)	H (Hartree)	G(T) (Hartree)	qh-G(T) (Hartree)
1	1	0.8655	35.57	-993.943240	0.274418	-993.647487	-993.722678	-993.717100
	2	0.7850	61.24	-993.988158	0.279212	-993.689260	-993.760967	-993.755715
	3	0.3001	56.50	-994.013187	0.281487	-993.713355	-993.779170	-993.775948
2	1	0.8835	35.02	-677.549588	0.332449	-677.197937	-677.265108	-677.262000
	2	0.7830	49.50	-677.572546	0.335977	-677.218424	-677.285767	-677.281396
	3	0.3817	60.99	-677.589464	0.338848	-677.233884	-677.297288	-677.293643
3	1	0.7190	46.87	-852.450664	0.400788	-852.026430	-852.099506	-852.097533
	2	0.7718	56.66	-852.490311	0.404021	-852.064150	-852.134397	-852.132488
	3	0.6411	56.99	-852.549563	0.407272	-852.121107	-852.190523	-852.188037

11. Correlation of RSS metric and relative bimolecular rates of radical decomposition.

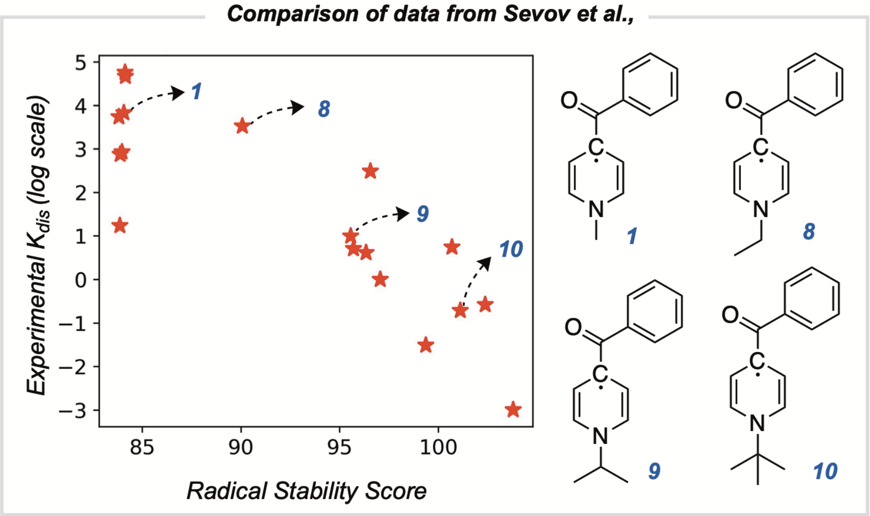

Fig. S8. Comparison of log(k_{rel}) and RSS for 18 radicals originally studied by Sevov (*J. Am. Chem. Soc.* 2017, 139, 8, 2924–2927). Buried volumes were generated at the N atoms.

12. RSS metrics generated with different radii used for buried volume analysis.

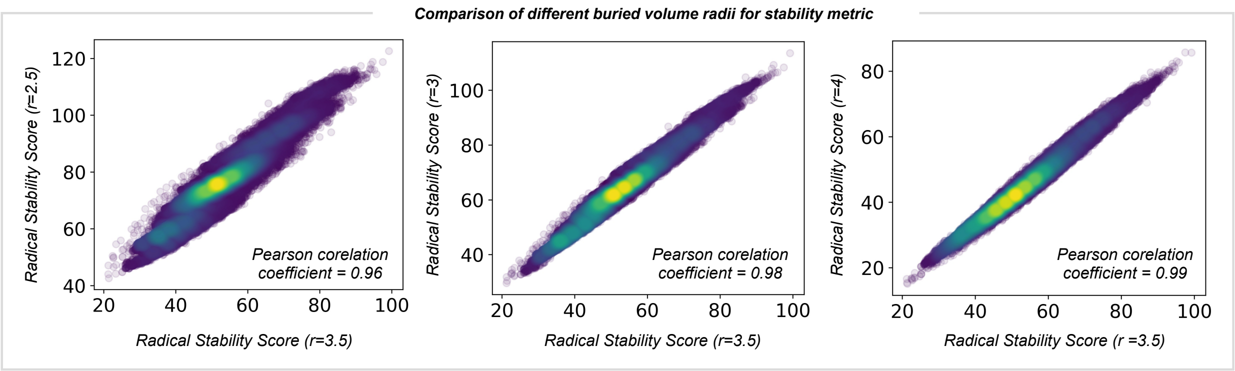

Fig. S9. Comparison of RSS metric generated with radii at 2.5, 3.0 and 4.0 Å against the more traditional value of 3.5 Å used for buried volume calculations.
